# Transcranial random noise stimulation (tRNS): a wide range of frequencies is needed for increasing cortical excitability

**DOI:** 10.1038/s41598-019-51553-7

**Published:** 2019-10-22

**Authors:** Beatrice Moret, Rita Donato, Massimo Nucci, Giorgia Cona, Gianluca Campana

**Affiliations:** 1 Dipartimento di Psicologia Generale, University of Padova, Via Venezia 8, 35131 Padova, Italy; 2 Human Inspired Technology Research Centre, University of Padova, Via Luzzati 4, 35121 Padova, Italy

**Keywords:** Excitability, Motor cortex

## Abstract

Transcranial random noise stimulation (tRNS) is a recent neuromodulation protocol. The high-frequency band (hf-tRNS) has shown to be the most effective in enhancing neural excitability. The frequency band of hf-tRNS typically spans from 100 to 640 Hz. Here we asked whether both the lower and the higher half of the high-frequency band are needed for increasing neural excitability. Three frequency ranges (100–400 Hz, 400–700 Hz, 100–700 Hz) and Sham conditions were delivered for 10 minutes at an intensity of 1.5 mA over the primary motor cortex (M1). Single-pulse transcranial magnetic stimulation (TMS) was delivered over the same area at baseline, 0, 10, 20, 30, 45 and 60 minutes after stimulation, while motor evoked potentials (MEPs) were recorded to evaluate changes in cortical excitability. Only the full-band condition (100–700 Hz) was able to modulate excitability by enhancing MEPs at 10 and 20 minutes after stimulation: neither the higher nor the lower sub-range of the high-frequency band significantly modulated cortical excitability. These results show that the efficacy of tRNS is strictly related to the width of the selected frequency range.

## Introduction

Transcranial random noise stimulation (tRNS) is a non-invasive electrical stimulation of the brain whereby a weak alternating current oscillating at random frequencies is delivered through the scalp using a pair of electrodes.

The frequency band of tRNS can encompass a full range (typically from 0.1 to 640 Hz) or can be delivered at low- or high-frequency (lf-tRNS or hf-tRNS; by convention, respectively ranging from 0.1–100 Hz and 101–640 Hz)^1^.

It is a relatively recent brain stimulation technique but, in the last few years, its popularity has sharply increased. The modulatory effects of tRNS – mainly involving the high-frequency band – have been probed with different motor, sensory and cognitive tasks. Studies on sensory or perceptual processing showed, for example, that hf-tRNS can improve visual detection or discrimination^2–4^ and can enhance the perception of facial identity^5^ and facial expression of emotions^6,7^. Visual motion adaptation, on the other hand, has shown to be either attenuated or enhanced depending on the frequency band used^8^. Findings on cognitive abilities revealed that hf-tRNS is even able to enhance arithmetic skills and calculation^9–11^. In patients, hf-tRNS has been successfully applied for reducing pain in multiple sclerosis^12^ and for decreasing motor cortex excitability in Parkinson’s disease^13^, as well as for reducing depressive symptoms^14^ and improving negative symptoms in schizophrenia^15^. Both lf-tRNS and hf-tRNS have shown promising results in reducing tinnitus intensity and distress^16–18^. Last but not least, hf-tRNS has shown an advantage over other electrical stimulation techniques in boosting perceptual and motor learning^19–26^.

Despite the proliferation of studies probing the effects of tRNS on cognitive functions, only a few studies have investigated its fundamental principles of functioning and the impact of the various stimulation parameters such as stimulation intensity, stimulation duration and frequency band.

Studies on sensory processing found that only intermediate stimulation intensities can increase visual detection or discrimination, suggesting that the perceptual enhancement is based on the phenomenon of stochastic resonance^3,4^. At a neuronal level, tRNS is believed to operate on the kinetics of activation and inactivation of Na^+^ channels^1,27–29^.

As for the effect of stimulation duration, a minimum of 5 minutes hf-tRNS over the motor cortex is required to obtain a significant increase in cortical excitability lasting for the next 10 minutes^30^.

Furthermore, only a few studies investigated the effect of the frequency band selected for the stimulation. In the visual domain, Campana and colleagues^8^ found that, while hf-tRNS delivered bilaterally over visual areas V5/MT reduced the duration of motion adaptation, lf-tRNS increased it. In the motor domain, the effect of lf- vs hf-tRNS applied on the motor cortex was probed with motor evoked potentials (MEPs). Terney and colleagues^1^ found a consistent excitability increase after 10 minutes of hf-tRNS (with frequencies spanning from 101 to 640 Hz) lasting up to one hour, as measured through both physiological measures and behavioural tasks, but no effect of lf-tRNS (with frequencies spanning from 0.1 to 100 Hz). This result was partially confirmed by Laczò and colleagues: after 10 minutes of hf-tRNS over the motor cortex, they found an increase in excitability for the following 40 minutes after stimulation^31^.

Besides an arbitrary subdivision of the frequency spectrum into two frequency bands (lf-tRNS ranging from 0.1 Hz to 100 Hz, and hf-tRNS ranging from 101 to 640 Hz), the effect of other frequency ranges on cortical excitability is still unknown. In particular, whether it is well established that the whole hf-tRNS band is able to produce an increase in cortical excitability^1,31–33^, it is not clear if the whole frequency band used in hf-tRNS is necessary for inducing such a change or whether sub-ranges of the high-frequency band are sufficient to provide a reliable effect. Moreover, since the distinction between lf-tRNS and hf-tRNS is made by considering two frequency range having two different frequency width, it is possible that the null effect on MEP produced by lf-tRNS on cortical excitability^1^ was not due to the (low) frequency range, but to the (narrow) frequency width.

**Figure 1:**
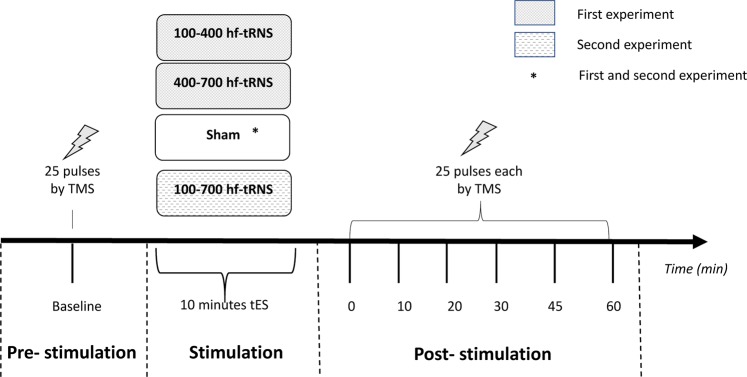
The experimental procedure for the four stimulation conditions is shown. 25 TMS-induced MEPs were recorded before tRNS (or Sham stimulation) and at each post-stimulation session.

To test this hypothesis, in Experiment 1 we divided the high-frequency band in two halves and compared the effects of these two sub-ranges: the first spanning 100–400 Hz and the second from 400 to 700 Hz. In this way, we only manipulated the frequency range, taking constant the frequency width. Since we did not find any relevant modulation of cortical excitability with either one of the two sub-ranges, in a second experiment (Experiment 2) we also tested the effect of the whole high-frequency band from 100 to 700 Hz (Fig. 1).

It is indeed possible that these two sub-ranges of frequency might modulate the brain activity differently. So far, however, no study has ever directly explored possible differential effects of hf-tRNS by considering both the spectra and the width of the frequency bands.

To investigate and compare the modulatory effects of these different spectra, we measured the amplitude variations of motor-evoked potentials (MEPs) induced by transcranial magnetic stimulation (TMS). This procedure is a well-established protocol and provides a reliable measure used to quantify the excitability changes induced in the primary motor cortex (M1)^1^.

## Results

A one-way repeated measures ANOVA showed no significant differences in MEP amplitudes between Stimulation conditions (Low-hf-tRNS, High-hf-tRNS, Sham) at baseline in Experiment 1 (F_2,10_ = 0.74, p = 0.49), and a paired t-test showed no significant differences between Stimulation conditions (Whole-hf-tRNS, Sham) at baseline in Experiment 2 (t_10_ = 1.16, p = 0.27). This implies that any differences between conditions arising from hf-tRNS could not be attributed to differences at baseline. MEP amplitudes were subsequently standardised using the mean and standard deviation of the baseline of each session.

### Model selection

**Figure 2:**
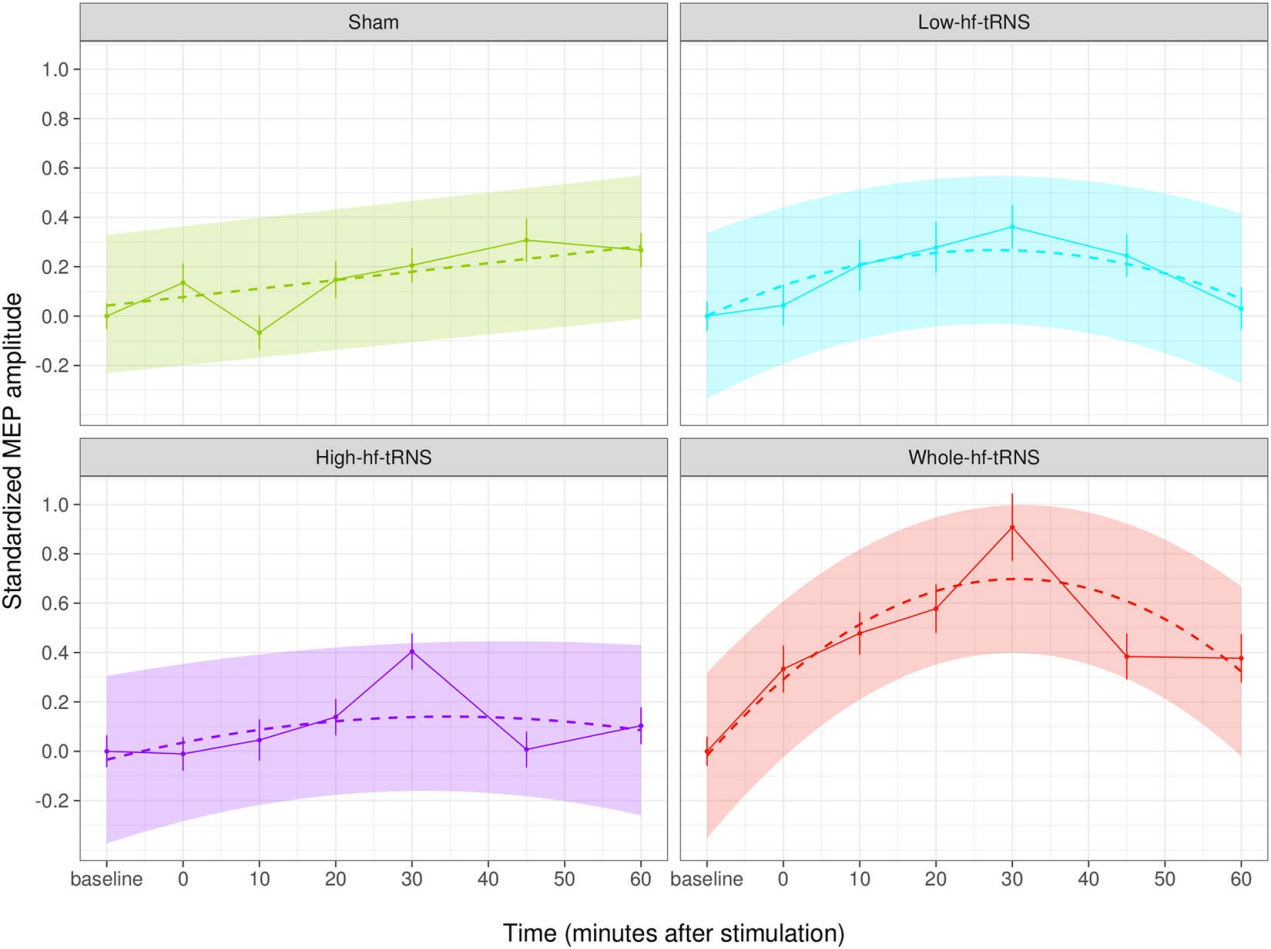
Effects plot for the predictors of the winner model (Time2 * Stimulation). Error bars represent standard error; coloured areas represents confidence bands.

In Table 1, the results of a nested mixed-effects model comparison on the data set of the two experiments are shown. In our analysis, only the quadratic effect of Time and the effect of Stimulation condition (Low-hf-tRNS, High-hf-tRNS, Whole-hf-tRNS, Sham) in interaction with the quadratic effect of Time significantly increased the prediction capacity, the latter being the winner model. More specifically, as also shown in Fig. 2, the amplitude of MEPs as a function of Time after tRNS can be described by an inverted U, but not for all Stimulation conditions. The sham condition does not show any quadratic trend but just a slight linear increase, whereas Low-hf-tRNS (100 Hz to 400 Hz) and High-hf-tRNS (400 Hz to 700 Hz) both have a mild curvature, compatible with a mild modulation of cortical excitability. Finally, Whole-hf-tRNS (100 Hz to 700 Hz) is the condition where the quadratic trend has more evidence, and the after-effects are more consistent and persistent.

**Table 1:** The result of model comparisons in a set of mixed-effects models on merged data set of the two experiments.

Fixed effects	Model df	AIC	BIC	Chisq	Chi Df	Pr(>Chisq)
	4	27164	27192			
Time	5	27163	27198	3.7131	1	0.054
Time^2^	6	27158	27200	6.1695	1	0.013*
Time^2^ + Stimulation	9	27161	27224	3.6572	3	0.301
Time^2^* Stimulation	15	27140	27245	32.2726	6	1.447e-05***

Df = degrees of freedom; AIC = Akaike’s information criterion; BIC = Bayesian information criterion; Chisq = chi-squared statistic; Chi Df = chi-squared degree of freedom; Pr(>Chisq) = probability value; participants are random effects in each model.

In order to pinpoint the differences between the conditions more in detail, data of the two experiments were also analysed separately.

### Experiment 1

**Figure 3:**
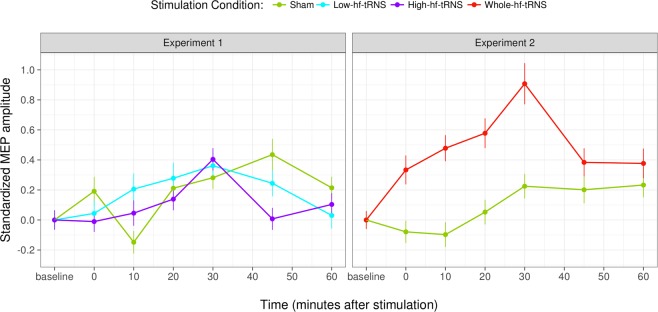
Results of Experiment 1 and 2. Standardised MEP amplitudes for different stimulation conditions at different time intervals from tRNS (or Sham stimulation); error bars represent standard error.

In Experiment 1, after Low-hf-tRNS (100 Hz to 400 Hz) or High-hf-tRNS (400 Hz to 700 Hz), a moderate and uneven increase in excitability is observable (Fig. 3, left panel). However, the ANOVA applied to the linear mixed effects model did not reach statistical significance either for the main effect of Stimulation condition (F_2,28.1_ = 0.14, p = 0.86) or for the main effect of Time (F_6,59.9_ = 1.22, p = 0.30). The interaction between Stimulation condition and Time indeed reached statistical significance (F_12,5487.4_ = 2.34, p = 0.005), but none of the twelve post-hoc comparisons between each level of tRNS and Sham at 0, 10, 20, 30, 45, 60 minutes was significant.

### Experiment 2

Experiment 2 shows a quite similar pattern. After Whole-hf-tRNS (100 Hz to 700 Hz), a marked increase of excitability is observable (Fig. 3 right panel). The two main effects were not significant (Stimulation condition: F_1,17.4_ = 2.91, p = 0.10; Time: F_6,60.8_ = 1.72, p = 0.13), but the interaction between Stimulation condition and Time did reach statistical significance (F_6,3683.9_ = 4.63, p < 0.001). According to the post-hoc analysis, differences between tRNS and Sham condition were significant at 10 (t_10_ = 2.47, p = 0.032) and 20 minutes (t_10_ = 3.06, p = 0.011) after stimulation.

Taken together these results show that, while stimulating with the whole frequency band of hf-tRNS produces a relevant increase in neural excitability that lasts up to twenty minutes after stimulation, shrinking the frequency range by half, and irrespectively of the specific (low or high) sub-range, sharply reduces the effects (if any) of stimulation.

## Discussion

The present study aimed to explore possible differential modulatory effects of hf-tRNS in cortical excitability as a function of the width of the selected frequency band. In particular, our goal was to understand better whether the increase in excitability of M1 neurons due to hf-tRNS is mainly due to the lower or the higher part of the frequency band. In Experiment 1 we thus split the entire high-frequency spectrum creating two new sub-ranges: 100–400 Hz and 400–700 Hz, and we compared the effect of these two sub-ranges of frequency to Sham stimulation. In Experiment 2, we tried to replicate the results of Terney and colleagues^1^ delivering the whole high-frequency band (100–700 Hz) and comparing the effects with those obtained with Sham stimulation (but note that Terney and colleagues^1^ used a slightly narrower frequency range, spanning from 101 to 640 Hz).

Unexpectedly, the results of Experiment 1 indicated that Low-hf-tRNS or High-hf-tRNS produced only a very mild (if any) modulation of cortical excitability. This variation was captured both by a quadratic effect of Time in the model comparison with the combined data of the two experiments and by a significant interaction between Time and Stimulation condition with data of Experiment 1, although no significant differences were found in post-hoc t-tests between any of the two tRNS conditions and Sham stimulation.

The results of Experiment 2, instead, showed a much more marked modulation of cortical excitability produced by Whole-hf-tRNS, as highlighted in Fig. 3 (right panel). This noticeable inflexion was confirmed both by the interaction between Stimulation condition and the quadratic effect of Time in the model comparison with the combined data of the two experiments and by the significant interaction between Time and Stimulation condition with data of Experiment 2. Here, post-hoc t-tests revealed that a significant difference between Whole-hf-tRNS and Sham was reached at 10 and 20 minutes after stimulation.

The first substantial result is that by splitting the high-frequency band of tRNS into two halves, a very tiny effect (if any) on cortical excitability is obtained. Neither the lower half nor the higher half of the high-frequency band seems able to have a substantial impact on cortical excitability. By reducing the range of frequencies, we also reduce the amount of noise (e.g. maximally shrinking the frequency range we are left with a single frequency, removing all the noise). This, in turn, might reduce the effect of tRNS in cortical excitability.

Drug studies show that tRNS effects on cortical excitability are sodium channels dependent^27^; also, there is neurophysiological evidence that *in vitro* random noise stimulation of rat’s neurons produce a faster reopening of Na^+^ channels with effects on both peak latency and amplitude of Na^+^ current^28,29^. Based on these findings we can speculate that the noisy fluctuations of the current produced by hf-tRNS could increase cortical excitability by decreasing the latency and increasing the peak amplitude of the Na^+^ current entering the cell, thus starting membrane’s depolarization^1,19,27–29^.

The optimal modulation occurs for intermediate levels of intensity^3,4,28^, but it is not clear what is the optimal amount of noise in terms of the frequency range, except for the fact that high-frequencies are needed^1^. Here we show that reducing (halving) the (high) frequency range, and thus reducing the noise, strongly impairs the modulatory effect of hf-tRNS on cortical excitability. It is possible that a lower amount of noise is not able to produce the same modulation of opening and closing of Na^+^ channels.

Similar to previous studies^1,31,33^, hf-tRNS has been able to enhance MEP amplitudes in post-stimulation measurements. However, unlike these studies, here we have been able to reliably increase cortical excitability only up to 20 minutes after stimulation. Compared to the 60 minutes found by both Terney and Moliadze and colleagues^1,33^, or the 40 minutes found by Laczò and colleagues^31^, our modulation of cortical excitability was shown to be much shorter.

Differences in the duration of modulation of cortical excitability might depend on many parameters of the electrical stimulation such as current type, current intensity, duration of stimulation, stimulation site, frequency range (for tRNS).

The main differences between the present and previous studies are stimulation intensity and ISI. Here, an ISI of 10 seconds has been used instead of 4-5 seconds as in other studies^1,32,33^. With this frequency of TMS pulses, even with Sham stimulation cortical excitability seems to have a slight, although non-significant, increase across successive blocks (Fig. 2, first panel). However, even if there was such a linear increase, this is unlikely to interact with the effect (if any) of tRNS. For what concerns stimulation intensity, the one used in the present study (1.5 mA) may exceed the optimal intensity for modulating cortical excitability in terms of a persistent enhancement. Both Terney and Moliadze and colleagues^1,33^ have successfully used 1 mA hf-tRNS in order to modulate MEP amplitudes up to 60 min, and studies on perceptual mechanisms have found an optimal enhancement of performance with 1 mA, while further increasing the intensity of stimulation worsened performance^3,4^. Laczò and colleagues^31^, on the other hand, have used an even higher intensity (2 mA) of hf-tRNS and found a relatively persistent increase in cortical excitability (approximately lasting twice with respect to the effect of the Whole-hf-tRNS found in the present study, but with a quadratic trend similar to that found in the present study). However, two issues could have reduced the amount of current reaching the target area in the study of Laczò and colleagues^31^. First, the fact that tRNS was applied over a different cortical site. It is well known that the distance between the cortical surface and the skull varies greatly depending on skull position. Since the site stimulated by Laczò and colleagues^31^ was much closer to the sagittal midline where there is a more considerable distance between the skull and the cortex, it is likely that more of the current was dispersed into the cerebrospinal fluid and less current arrived at the target location. Second, the size of the electrodes used by Laczò and colleagues^31^ was more than double the size used by Terney and colleagues^1^ and in the present study. Both factors have likely decreased the amount of current reaching the target area, thus counterbalancing the high intensity used in that study.

In conclusion, our data support the suggestion that an intermediate intensity of tRNS is optimal in increasing cortical excitability for a prolonged interval, whereas higher intensities can reduce this effect. The novel finding is that a large amount of noise (i.e. a wide range of frequencies) is needed to produce a significant and persistent increase in cortical excitability, while a smaller amount of noise (i.e. a narrower frequency range) does not elicit such a modulatory effect. This finding questions the assumption that the differential effects of lf-tRNS and hf-tRNS are due to the low- vs high-frequency bands, where in fact they could be due to the fact that they encompass respectively narrow (lf-tRNS, spanning about 100 Hz) and wide frequency ranges (hf-tRNS, spanning between 500 and 600 Hz).

These results should be carefully taken into consideration when tRNS is used in protocols aiming to improve brain functions; mainly, the suggestion is to favour, besides moderate intensities as suggested by other studies^3,4^, also wider frequency ranges, that seem to yield a more pronounced effect.

## Material and Methods

### Participants

A total of 14 healthy female students (mean age 21, range 19–25 years) of the University of Padova, took part in this study. More specifically, 8 out of 14 participants took part in both experiments, and 3 out of 14 participated in Experiment 1 or 2 only, so to obtain a group of 11 participants for each experiment. All the participants had no TMS contraindications^34^ assessed through a written questionnaire, and gave written informed consent according to the Declaration of Helsinki. All participants were right-handed (assessed by the Edinburgh Handedness Inventory)^35^. This study was approved by the local Ethics Committee (Comitato Etico della Ricerca Psicologica (Area 17) of the University of Padova, Protocol Number: 2459). All participants tolerated the stimulation protocol well, and no side-effects were reported.

One limitation of this study is that we only tested female participants. However, it is reasonable to assume that these results can be generalized to male participants or to gender-balanced samples. In fact, although cortical excitability of male and female participants is only similar during the follicular phase of the menstrual cycle^11,36,37^, aim of this study was not to measure absolute values of MEPs but rather the amount of change in excitability due to tRNS with respect to a common MEP baseline (~1 mV) that was measured at every session^1^. Therefore, any inter-individual or intra-individual differences in MEP amplitude due to ovarian hormones or to any other cause (poor sleeping, caffeine intake, circadian rhythms, etc.) should be accounted for. Ovarian hormones could also affect the neural mechanisms underlying the effects of transcranial electrical stimulation. In fact, a prolonged aftereffect of tDCS in females with respect to males was found, but only in terms of a reduced excitability due to cathodal stimulation, whereas no differences were found with the increased excitability due to anodal stimulation^38^. Since both anodal tDCS and hf-tRNS produce an increase of cortical excitability, it is reasonable to assume that ovarian hormones do not alter the effect of transcranial electrical stimulation in either case.

### Apparatus

#### Transcranial random noise stimulation

The current was delivered by a battery-driven stimulator (BrainStim, EMS) using a pair of rubber electrodes covered by sponges soaked in saline solution. The target electrode was 16 cm^2^ large (current density: 0.09 mA/cm^2^), was positioned above the primary motor area (M1) and its centre matched the cortical representation of the first dorsal interosseous muscle (FDI) (see paragraph on “Localization of stimulation site and motor threshold”). The reference electrode, 60 cm^2^ large (current density: 0.02 mA/cm^2^), was placed above the contralateral orbitofrontal area. This position is widely used for positioning the reference electrode^1,27^.

In all conditions but Sham, tRNS was delivered for 10 minutes with a current intensity of 1.5 mA and 0 mA offset. Current linearly increased in intensity up to 1.5 mA during the first 30 s of stimulation. In the Sham condition, the current linearly increased for the first 15 s up to a 1.5 mA and then decreased to 0 mA in the next 15 s. The current density was maintained within the safety limits (i.e., below 1.0 A/m^2^)^39^.

#### Motor evoked potentials (MEPs)

Corticospinal excitability was assessed by measuring the amplitude of MEPs of the first dorsal interosseous (FDI) by TMS over M1 using a Magstim Rapid^2^ stimulator.

The stimulator was wired to a computer where a Matlab script triggered 25 pulses with an inter-stimulus interval (ISI) of 10 seconds, delivered through a 70 mm figure-of-eight coil.

Surface electromyogram (EMG) was recorded from FDI muscle of the right hand via Ag/Agl electrodes (the active electrode on FDI, the inactive one on the third phalanx of the index oh the same hand and the ground on the upper side of the wrist) in a belly-tendon montage. Using System PLUS Evolution software (Myohandy Matrix Line, Micromed) raw signals were amplified and digitised, setting a sampling rate of 2048 Hz and a bandpass filter of 5–600 Hz. The electrode impedance was kept below 10 kΩ. The epoch considered was 200 ms and a time window between 5 and 50 ms was recorded after the TMS pulse to obtain the difference between the maximum and the minimum peak, automatically detected by the software.

#### Localisation of stimulation site and motor threshold

For each participant, the stimulation site was selected in the following way: first, we found the point of the skull closest to the Talairach coordinates of the hand area^40,41^ using a frameless neuronavigation system (BrainSight 2.3.8 together with an NDI Polaris Vicra camera). Then, a 3 by 3 grid centred on the previously found site and with 1 cm distance between them was marked on the skull of each participant with small stickers. Each point of the grid was tested with single-pulse TMS starting from an intensity of 30% of the maximum stimulator output (MSO) and increasing it in steps of 5–10% until MEPs equal or above 1 mV were elicited. The stimulation site of the right FDI was identified as the point eliciting the largest MEP, TMS intensity being equal. Once the final stimulation site was found, its coordinates were recorded and maintained equal for each participant during all sessions, using the stereotactic frameless neuronavigation system^42^. The resting motor threshold was defined at each session as the intensity of TMS needed to evoke ~1 mV peak-to-peak MEP amplitude. It was assessed with single-pulse TMS by increasing or decreasing TMS intensity (1–2%) till reaching the target of ~1 mV peak-to-peak MEP amplitude and verified with successive 10 pulses with 4 s of ISI.

### Experimental procedure

Two different experiments have been run in this study. The time interval between the two experiments was about 2 months, whereas the interval between sessions (within the same experiment) was 1–3 days. The procedure is schematically shown in Fig. 1.

Participants were seated in a comfortable chair with a mounted headrest throughout the experiments.

25 MEPs using single-pulse TMS were recorded at baseline (immediately before stimulation) and after tRNS at 0 min, 10 min, 20 min, 30 min, 45 and 60 min after stimulation. Since consecutive TMS pulses with short ISI might affect MEP amplitudes, we set 10 seconds ISI to reduce any potential interference^43^. The coil was positioned around 45-degree rotation about the parasagittal plane to induce a posterior-to-anterior current in the underlying cortex.

The order of the stimulation conditions was counterbalanced within participants, with at least 2 days between sessions:

Experiment 1 comprised three tRNS sessions:

- Low-hf-tRNS (L- hf-tRNS) with frequency ranging from 100 Hz to 400 Hz
- High-hf-tRNS (H- hf-tRNS) with frequency ranging from 400 Hz to 700 Hz
- Sham stimulation

Experiment 2 consisted of two tRNS sessions:

- Whole-hf-tRNS (W-hf-tRNS) with frequencies ranging from 100 Hz to 700 Hz
- Sham stimulation

Participants were blind towards the experimental conditions and were not able to distinguish between real and Sham stimulation.

### Analysis and statistics

MEP amplitude was automatically calculated by System PLUS Evolution software (Myohandy Matrix Line, Micromed). For each experiment, MEP amplitudes of each Stimulation condition at baseline (before stimulation) were compared: with a one-way repeated measures ANOVA in Experiment 1, and with a paired t-test in Experiment 2. Since no significant differences were found between any of the Stimulation conditions at baseline, all MEP amplitudes were standardised using the mean and standard deviation of the baseline of each session.

In order to have an overview of the data, results of Experiment 1 and 2 were combined, and a mixed effect regression was run comparing a set of nested mixed-effects models^44^ with Stimulation condition (tRNS: Low-hf-tRNS, High-hf-tRNS, Whole-hf-tRNS, Sham) and Time (before, 0, 10, 20, 30, 45, 60 min post-stimulation) as fixed effects, with Participant, nested in Stimulation condition and Time as random effects. Nested models are a succession of models in which, starting from a null model, the successive one contains all the terms of the previous with one additional term. On Nested models it is possible to compare the prediction capacity of each model (Akaike’s information criterion – AIC – and Bayesian information criterion – BIC) with that of the previous one, controlling for the variations due to chance (p value). A stepwise ANOVA for model selection (lowest AIC value and p-value) was used to identify the combinations of variables that best predicted the outcome variabilities. An effects plot^45^ of the winner model was implemented.

Then, data of the two experiments were also analysed separately.

For Experiment 1, Type III Analysis of Variance with a linear mixed effects model and Satterthwaite’s approximation of degrees of freedom was applied^46^. Fixed effects were Stimulation condition (tRNS: Low-hf-tRNS, High-hf-tRNS, Sham) and Time (before stimulation, 0, 10, 20, 30, 45, 60 minutes post-stimulation]; random effects were Participant nested in Stimulation condition and Time. Similarly, for Experiment 2, Type III Analysis of Variance with a linear mixed effects model and Satterthwaite’s approximation of degrees of freedom was applied with Stimulation condition (Whole-hf-tRNS, Sham) and Time (before stimulation, 0, 10, 20, 30, 45, 60 min post-stimulation) as fixed effects and Participant nested in Stimulation condition and Time as random effects. Student’s t-test was used to compare MEPs in a post-hoc analysis. Effects were considered significant with p < 0.05.

## Data Availability

The datasets generated and/or analysed during the current study are available from the corresponding author on reasonable request.
